# Are we ready to use hypofractionated instead of conventional radiotherapy for prostate cancer? Not yet

**DOI:** 10.1590/S1677-5538.IBJU.2018.0734

**Published:** 2019

**Affiliations:** 1 Departamento de Radioterapia, Hospital AC Camargo Cancer Center

Prostate cancer is still the most frequently diagnosed cancer in American men despite a 7.6% decrease in cancer incidence between 2011 and 2014 (1). In Brazil, about 68,220 men are expected to be diagnosed with prostate cancer in 2018 (2).

Recently, a consensus on the use and indications of Hypofractionated Radiation Therapy (hRT) or Ultrahypofractionation radiotherapy, also referred to “extreme hypofractionation”, “stereotactic ablative body radiation therapy” and “stereotactic body radiation therapy” (SBRT) for Localized Prostate Cancer (PCa) from the ASTRO, ASCO, and AUA were published (3-5).

This consensus was based on key questions that addressed the indications of the different schedules of hypofractionation for the different risk groups of PCa when compared to conventionally fractioned external beam radiotherapy (EBRT), using daily fractions of 180-200 cGy given in 7 to 8 weeks, for doses up to 7,800 cGy.

Several studies have already provided evidence for the efficacy of dose-escalation on biochemical control (BC) of PCa and results from randomized trials (RCTs) have shown a direct relation between increasing the radiation dose given to the prostate and/or seminal vesicles and BC (6-9); however, randomized data comparing different methods of dose escalation are sparse (10).

Unfortunately the consensus on the use of hRT did not include forms of hypofractionation that combine different techniques of radiation like EBRT associated to brachytherapy, using either high or low dose rate sources. The role of high dose rate (HDR) brachytherapy in the treatment of men with PCa is not well defined, but the results of the trials mentioned above have shown that escalated doses are superior to conventional doses to achieve BC in all risk groups of PCa. HDR brachyteharpy can escalate the dose given to the prostate by the combination with EBRT and has also the potential biological advantage through the delivery of doses in higher levels than the ones evaluated in the published consensus (11). Mature data published have already evaluated the 10-year outcomes of intermediate- and high-risk patients noting a clear dose response by increasing the dose escalation through HDR doses (12). The results of the first randomized prospective trial addressing dose escalation using an HDR and EBRT were published in 2012, noting 18% increase in the disease specific survival for patients who had combined modality treatments (p = 0.04), reflecting a 31% reduction in the risk of recurrence (p = 0.01) and no evidence of an increase in long-term severe morbidity (13).

Moderate hRT was defined in that guideline as treatments given with fractions size between 240 cGy and 340 cGy per day, three to five times a week over 3.8 to 5.6 weeks. SBRT was defined as EBRT administered with fractions size of more than 500 cGy independent of considerations of technique used.

The literature has four large prospective RCTs and additional single institution RCTs demonstrating that hRT provides BC that is similar to EBRT. It is also important to point out that, despite a limited follow-up beyond five years for most RCTs, a small increased risk of acute gastrointestinal toxicity is observed, but with similar late gastrointestinal risk. No additional acute and late genitourinary toxicity with hRT was noted (14-23).

It is important to highlight the fact that the optimal radiation regimen still cannot be determined since most of the multiple fractionation schemes evaluated in clinical trials have not been compared in parallel. Regimens of 6,000 cGy and 7,000 cGy, given in 20 and 28 daily fractions, respectively are the most frequent found in the literature.

Information regarding dose constraints is also conflicting, but three trials published reported constraints for bladder and rectum on solid contours (9, 24, 25).

To date, there are no published efficacy and toxicity data from RCTs comparing SBRT and conventionally fractionated EBRT, nor specific normal tissue constraints. Most of published results apply to patients with prostate volumes up to 100 cm3 and with mild to moderate urinary symptoms at baseline. Doses of 3,500 to 3,625 cGy given in 5 fractions of 700 to 725 cGy are recommended, and on plan evaluations at least two dose-volume constraint points for rectum and bladder shall be used (26, 27).

The quality of evidence of each recommendation statement was categorized on the grade guidelines published by Balshem et al. (28) as high, moderate, low, or very low, indicating:

- “High: the panel was very confident that the true effect lies close to that of the estimate of the effect;
- Moderate: the panel was moderately confident in the effect estimate: The true effect is likely to be close to the estimate of the effect, but there is a possibility that it is substantially different;
- Low: the panel confidence in the effect estimate is limited: The true effect may be substantially different from the estimate of the effect;
- Very Low: the panel has very little confidence in the effect estimate: The true effect is likely to be substantially different from the estimate.”

The following questions were addressed;

Key Question 1 – hRT is indicated for the following risk groups of PCa.

1A - For low-risk prostate.
  - Recommendation strength: Strong
  - Quality of evidence: High
  - Consensus: 100%
1B - For intermediate-risk prostate.
  - Recommendation strength: Strong
  - Quality of evidence: High
  - Consensus: 100%
1C - For high-risk prostate (not receiving pelvic lymph nodes irradiation).
  - Recommendation strength: Strong
  - Quality of evidence: High
  - Consensus: 94%

Key Question 2 – Men should be counseled about the small increased risk of acute gastrointestinal (GI) toxicity with hRT?

- Recommendation strength: Strong
- Quality of evidence: High
- Consensus: 100%

Key Question 3 – Regarding patient age, associated comorbidity, anatomy, or urinary function and regimens of 60 Gy and 70 Gy, given in 20 and 28 daily fractions.

3A – The optimal regimen cannot be determined.
  - Recommendation strength: Conditional
  - Quality of evidence: Moderate
  - Consensus: 100%
3B – one moderately hypofractionated regimen is not suggested over another and hRT regimens do not appear to be impacted by patient age, comorbidity, anatomy, or urinary function.
  Recommendation strength: Conditional
  - Quality of evidence: Moderate
  - Consensus: 100%

Key Question 4 – SBRT and risk groups of PCa.

4A - for low-risk prostate SBRT may be offered as an alternative to conventional fractionation.
  - Recommendation strength: Conditional
  - Quality of evidence: Moderate
  - Consensus: 88%
4B - for intermediate-risk prostate SBRT may be offered as an alternative to conventional fractionation.
  - Strength of recommendation: Conditional
  - Quality of evidence: Low
  - Consensus: 94%
4C - for high-risk prostate SBRT may be offered as an alternative to conventional fractionation.
  - Strength of recommendation: Conditional
  - Quality of evidence: Low
  - Consensus: 94%

Key Question 5 - SBRT may be offered to low- and intermediate-risk patients with prostate sizes less than 100 cm^3^.

- Recommendation strength: Conditional
- Quality of evidence: Moderate
- Consensus: 88%

Key Question 6 - regarding normal tissues constraints.

Statement: At least two dose-volume constraint points for rectum and bladder should be used for hRT or SBRT: one at the high-dose end (near the total dose prescribed) and one in the mid-dose range (near the midpoint of the total dose).
  - Recommendation strength: Strong
  - Quality of evidence: Moderate
  - Consensus: 100%

Key Question 6 – the associated margin definitions for the target.

Most commonly reported margins describe an isotropic 5 mm expansion around the CTV with the exception of a 3 mm posterior expansion. So, it is not recommended to use margins that deviate from those already published and used as references in the consensus.
  - Recommendation strength: Strong
  - Quality of evidence: Low
  - Consensus: 100%

Key Question 7 – Image guided radiotherapy (IGRT) should be universally recommended when delivering hRT or SBRT.

- Recommendation strength: Strong
- Quality of evidence: Moderate
- Consensus: 100%

Key Question 8 – Non-modulated techniques are not recommended when delivering hRT or SBRT.

- Recommendation strength: Strong
- Quality of evidence: Moderate
- Consensus: 100%
